# From orchard to table: Insight into the dissipation, metabolism, processing and risk assessment of 14 pesticides in sweet cherry

**DOI:** 10.1016/j.fochx.2026.104659

**Published:** 2026-10-19

**Authors:** Kai Cui, Liping Fang, Ruiyan Ding, Wanru Su, Qiang Liu, Shuai Guan, Jingyun Liang, Jian Wang, Teng Li, Zhan Dong

**Affiliations:** a Institute of Agricultural Products Quality Safety and Standards, Shandong Academy of Agricultural Sciences; Shandong Provincial Key Laboratory of Test Technology on Food Quality and Safety, Jinan, Shandong 250100, People's Republic of China; b College of Plant Protection, Shandong Agricultural University, Tai'an, Shandong 271018, People's Republic of China; c Lunan Pharmaceutical Group Co. Ltd., Linyi, Shandong 276006, People's Republic of China

**Keywords:** Pesticide residue, Cherry, Dissipation, Processing factor, Risk assessment

## Abstract

This study provides a comprehensive investigation into the dissipation, metabolism, processing factors (PFs) and risk assessment of 14 commonly used pesticides in cherries from field to processing. Field trials indicated that the dissipation of all pesticides followed first-order kinetics, with half-lives ranging from 3.11 to 21.66 days. Metabolites carbendazim and clothianidin were detected at concentrations of 2.24–443.05 μg/kg. Washing reduced pesticide residues to varying degrees, yielding PFs of 0.10–1.23 (mostly <1), and showed a negative correlation with washing duration. In contrast, drying processes concentrated residues, with PFs of 0.75–3.93 for microwave drying and 2.99–6.70 for oven drying. Oven drying resulted in generally higher residue levels compared to microwave drying. Dietary risk assessment revealed that chronic and acute exposure risks from pesticide residues in cherries remained within acceptable thresholds for children and adults. These results offer practical guidance for the safe consumption of cherries and contribute to evidence-based food safety management.

## Highlights

• Residue fate of 14 pesticides was studied in cherries from orchard to table.
• The half-lives of pesticides in cherries were 3.11–21.66 days.
• Key metabolites detected included carbendazim and clothianidin.
• An up to 6.70-fold concentration of residues was observed during drying.
• Human health risks were within acceptable limits.

## Graphical abstract

**Figure:**
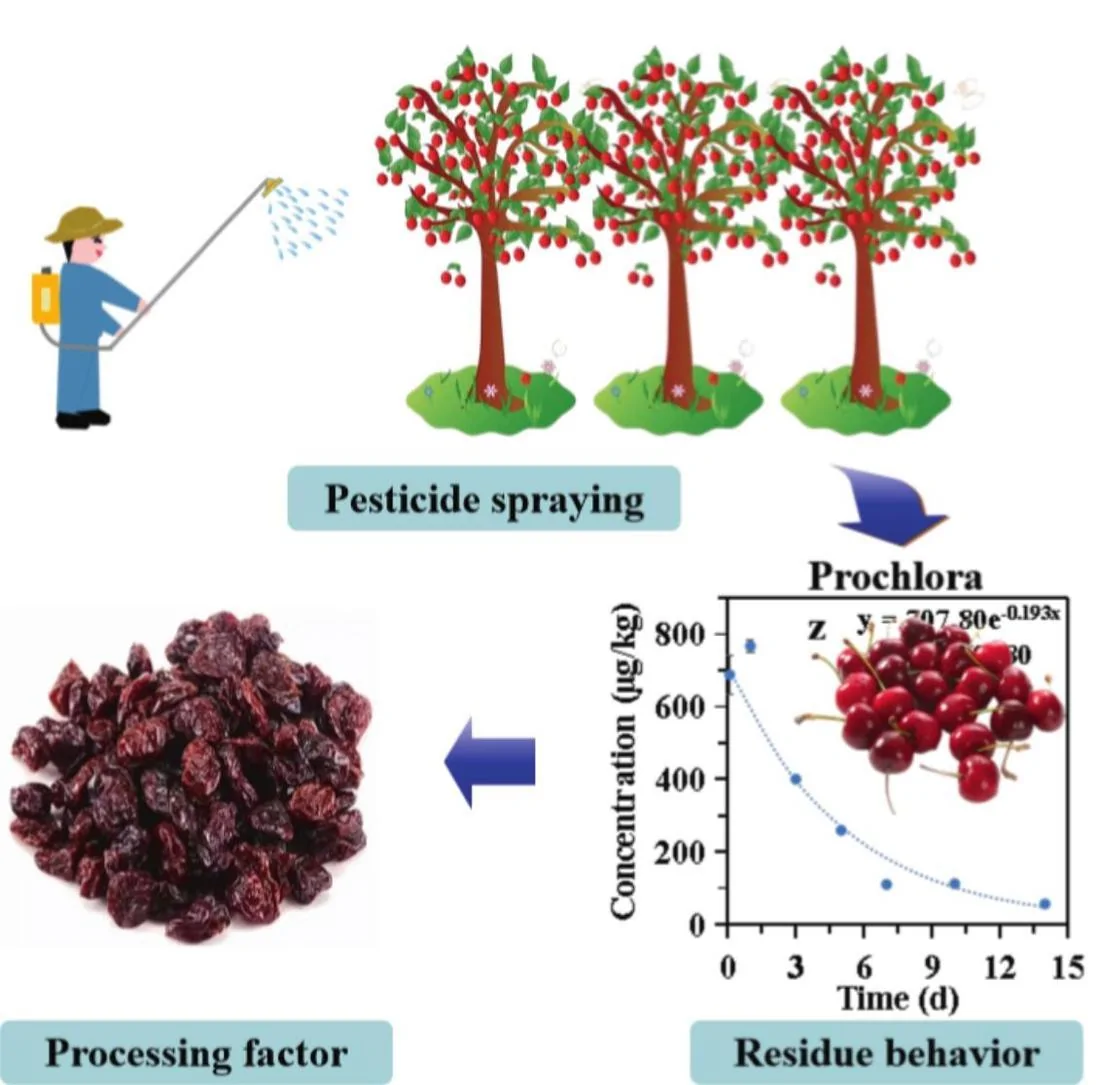

## 1 Introduction

Cherries (*Prunus avium* L.) are among the most widely consumed and economic fruits worldwide owing to their exceptional sensory quality, nutritional value and high contents in bioactive compounds, such as anthocyanins, carotenoids, polyphenols, organic acids and vitamins (Gonçalves et al., 2024; Wang et al., 2025). According to more recent statistics, from 2018 to 2023, the cultivation area of cherries in China has remained stable at approximately 8500 ha, with an annual production of around 35,000 metric tons (https://www.fao.org/faostat/en/#data/QCL/visualize). However, cherry cultivation is highly susceptible to a range of pests, such as cherry fly, spotted wing drosophila, black cherry aphid and red spider mite, as well as fungal diseases including brown rot, shot hole and leaf spot (González-Núñez et al., 2022; Noble et al., 2023). Without effective control measures, these threats can lead to substantial economic losses. To safeguard both the quality and yield of cherry production, growers routinely employ a range of pesticides to manage various diseases and pests (Balkan et al., 2025). However, the adverse effects of pesticides on human health have long been a subject of ongoing concern and discussion. Therefore, investigating the residue levels and dissipation behavior of pesticides in cherries is of significant importance for safeguarding human health.

Research on pesticide residues in cherries has primarily focused on the monitoring of market samples. These studies have consistently documented the pervasive presence of various pesticides, including some exceeded the maximum residue limits (MRLs) on cherries (Gormez et al., 2022; Jankowska et al., 2024; Łozowicka et al., 2025; Orecchio et al., 2026; Szpyrka et al., 2015). Several field-based investigations have examined the dissipation behavior and conducted risk assessments of specific pesticides in cherry cultivation (Balkan et al., 2025; Hasanzadeh Khorram et al., 2024; Yao et al., 2021). However, the scope of these studies remains limited to a small number of pesticides, which could not comprehensively reflect the risk of pesticide residues in cherries. In practice, a wide array of pesticides is applied throughout the growth cycle of cherries to manage diverse pests and diseases. Understanding the residue levels and dissipation kinetics of pesticides is crucial for assessing their persistence and forecasting residue changes in cherries (Cui et al., 2025; Fantke & Juraske, 2013). This understanding is fundamental to informing rational pesticide application and ensuring food safety. Therefore, it is essential to expand investigating efforts to include all commonly used pesticides and comprehensively evaluate their residue behavior and potential health risks to ensure safe application practices.

Cherries are typically consumed fresh to maximize retention of their natural flavor and nutritional density (Blando & Oomah, 2019; Gonçalves et al., 2024). Prior to consumption, washing is recommended to remove surface contaminants; however, this process may also modify pesticide residue profiles, potentially altering dietary exposure risks (Li, Liu, et al., 2024; VanWoerkom et al., 2022). Despite the unique organoleptic qualities–such as a balanced sweet-tart flavor and firm, juicy texture–that are best appreciated in fresh cherries, their high moisture content and delicate structure make them highly perishable. These characteristics contribute to limited shelf life and pose significant challenges for postharvest preservation (Krishnan et al., 2025; Liu et al., 2025). In recent years, the processing of cherries into dried products has gained popularity as a method to concentrate their nutritional content and prolong their shelf life (Jawad et al., 2025; Uribe et al., 2023). However, the drying process may cause pesticide residues to become concentrated in the final dried products (Cui et al., 2024; Hrynko et al., 2023; Hu et al., 2025), potentially leading to a higher risk of dietary exposure. Given the significant influence of processing on pesticide residue levels in cherries, it is imperative to investigate their effects to facilitate an accurate human health risk assessment.

This study presents the first systematic investigation into the dissipation kinetics, metabolism, processing factors (PFs) and dietary risk assessment of 14 pesticides in cherries across the whole supply chain from orchard planting to final consumption. Target pesticides were selected based on their registration for cherry cultivation in China and frequent detection in routine stone fruit residue monitoring. Field trials were first conducted to evaluate the residual levels and dissipation behavior of these pesticides in cherries. Subsequently, field-collected samples were subjected to washing and drying treatments to examine changes in pesticide residues during processing. The key objectives were to (1) elucidate the dissipation patterns of the target pesticides in cherries, (2) quantify the PFs associated with washing and drying treatments and (3) evaluate the dietary risk posed by these pesticides to humans from cherry consumption. The findings offer a comprehensive understanding of the behavior of commonly applied pesticides from field through processing, and support evidence-based recommendations for the safe and rational use of these pesticides in cherry production.

## 2 Materials and methods

### 2.1 Chemicals and reagents

Individual standards of 14 pesticides–namely acetamiprid, difenoconazole, fluopyram, imidacloprid, kresoxim-methyl, mefentrifluconazole, prochloraz, pyraclostrobin, pyridaben, spirodiclofen, tebuconazole, thiamethoxam, thiophanate-methyl and trifloxystrobin–as well as three metabolites (acetamiprid-*N*-desmethyl, carbendazim and clothianidin) were procured from Alta Scientific Co., Ltd. (Tianjin, China). The physicochemical properties of these pesticides are provided in Table S1. These standards were prepared as stock solutions in acetonitrile at concentrations of 1000 mg/L or 100 mg/L. High-performance liquid chromatography (HPLC)-grade acetonitrile and formic acid were sourced from Fisher Scientific (Geel, Belgium) and Macklin Biochemical Co., Ltd. (Shanghai, China), respectively. Analytical-grade sodium chloride (NaCl) and anhydrous magnesium sulfate (MgSO_4_) were obtained from Sinopharm Chemical Reagent Co., Ltd. (Shanghai, China). Additionally, nylon syringe filters (0.22 μm) and octadecylsilane (C18) were supplied by Agela Technologies (Beijing, China).

### 2.2 Field trials and sample collection

Field trials were carried out during the cherry cropping season of 2025 under open-field conditions in Changqing District, Jinan City, Shandong Province, China. The experimental setup included two plots: a treatment plot (comprising six trees) and a control plot (also six trees). Each pesticide was applied at the maximum recommended dosage and times, with detailed parameters provided in Table S2. The pesticides were thoroughly mixed into water and sprayed at 7-day intervals. For the dissipation study, cherry samples (>1.0 kg) were collected at 2 h and 1, 3, 5, 7, 10 and 14 days after the final application. Corresponding fruit phenological stages were determined according to the Biologische Bundesanstalt, Bundessortenamt und Chemische Industrie (BBCH) scale, with assigned BBCH codes of 81, 81, 85, 85, 85, 87 and 89, respectively. For the processing study, a separate batch of cherries (>10.0 kg) was sampled 1 day (BBCH 81) after the last spray. All samples were immediately homogenized or processed upon delivery to the laboratory.

### 2.3 Cherry processing

Washing treatment: Approximately 3.0 kg of cherries were immersed in tap water (25 °C) and manually washed. Samples were collected after different washing durations: 1, 2, 5 and 10 min.

Drying treatment: All cherry samples were initially pitted. The fresh fruits were subsequently dried into dried cherries using two methods: oven drying and microwave drying. For oven drying, 3.0 kg of cherries were dried in an oven at 60 °C for 12 h. For microwave drying, 3.0 kg of cherries were processed in a microwave oven at 700 W for 20 min. During this process, the liquid was drained at five-minute intervals, with thorough remixing performed between each drainage step.

After processing, each sample was homogenized and stored at −20 °C until analysis.

### 2.4 Sample preparation and instrumental analysis

The Quick, Easy, Cheap, Effective, Rugged and Safe (QuEChERS) method, as originally described by Anastassiades et al. (2003), was employed with minor modifications for the extraction of pesticide residues from cherry samples. Briefly, homogenized samples (5 g fresh or 2 g dried weight) were weighed into 50 mL Teflon centrifuge tubes. Each tube was then supplemented with 10 mL of acetonitrile and vigorously shaken for 5 min. Following this, 3.0 g of NaCl was added, and the mixture was shaken for an additional 5 min before being centrifuged at 4000 rpm for 5 min. A 5.0 mL aliquot of the supernatant was subsequently transferred to a purification tube containing 150 mg of C18 sorbent and 900 mg of anhydrous MgSO_4_, followed by shaking for 1 min. After centrifugation at 4000 rpm for 5 min, 1 mL of the purified supernatant was passed through a 0.22-μm nylon filter prior to analysis via HPLC-tandem mass spectrometry (HPLC–MS/MS) analysis.

The analysis was conducted using HPLC–MS/MS. The system consisted of an Agilent Technologies 1290 Infinity II HPLC interfaced with a 6495 MS/MS (Agilent, USA), operated in positive electrospray ionization (ESI+) mode. Chromatographic separation was achieved on a Poroshell 120 EC-C18 column (50 mm × 2.1 mm, 2.7 μm, Agilent, USA) maintained at 40 °C, with an injection volume of 5 μL. The mobile phase comprised (A) acetonitrile and (B) water containing 0.1% formic acid, delivered at a flow rate of 0.3 mL/min according to the following gradient program: 30% A (0–0.5 min), increased to 70% A (2 min), held until 6 min, returned to 10% A (6.5 min) and re-equilibrated until 8 min. The MS/MS was performed with the following source parameters: sheath gas temperature, 325 °C; gas temperature, 250 °C; sheath gas flow, 11 L/min; gas flow, 14 L/min; nebulizer pressure, 35 psi; capillary voltage, 4000 V; and nozzle voltage, 1500 V. All target compounds were analyzed in multiple-reaction monitoring mode, with detailed parameters listed in Table S3.

### 2.5 Method validation

In accordance with the European Commission SANTE/11312/2021 v2 guideline (Pihlström et al., 2021), the proposed method was comprehensively validated for target analytes. Key performance parameters–including linearity, limit of quantification (LOQ), matrix effect (ME), accuracy and precision–were evaluated. Prior to validation, a blank sample was analyzed to confirm the absence of all target pesticides, thereby establishing its suitability for use in the validation procedure. Linearity was assessed using both solvent-based and matrix-matched calibration curves, spanning concentration ranges of 1–1000 μg/L. The LOQ was defined as the lowest validated spiked concentration in cherries that satisfied all acceptable performance criteria. The ME indicates signal suppression or enhancement due to the cherry matrix. Accuracy and precision were evaluated through recovery studies performed at three concentration levels (1 or 5, 100 and 2000 μg/kg), each with five replicates.

### 2.6 Health risk assessment

In this study, chronic and acute dietary risk assessments associated with cherry consumption were conducted for children and adults, in accordance with the methodologies recommended by the FAO/WHO (2025). The chronic dietary risk was evaluated by calculating the estimated daily intake (EDI, μg/kg bw) and the corresponding chronic risk quotients (RQc) for each pesticide using Eqs. (1) and (2). The acute dietary risk assessment involved estimation of the short-term intake (ESTI, μg/kg bw) and the acute risk quotient (RQa), derived from Eqs. (3) and (4).

(1) EDI=STMR×F/bw/1000

(2) RQc=EDI/ADI

(3) ESTI=HR×LP/bw/1000

(4) RQa=ESTI/ARfD

In the equations above, STMR and HR (μg/kg) represent the median and highest residue levels detected in cherries, respectively. F refers to the average daily cherry consumption (0.04 g); bw denotes body weight (16.2 kg for children and 67.5 kg for adults); and LP corresponds to the large portion consumption of cherries (187.50 g for children and 740.52 g for adults). All dietary consumption data were sourced from FAO/WHO (2025). ADI and ARfD (μg/kg bw) refer to the acceptable daily intake and acute reference dose, respectively, as provided in Table S4. An RQc or RQa value exceeding 1 signifies an unacceptable risk to human health, whereas values <1 are considered acceptable. As specified in the documents from the Joint FAO/WHO Meeting on Pesticide Residues (JMPR), thiamethoxam and its metabolite clothianidin are treated as individual components within the residue definition for dietary risk assessment. By contrast, the residue definition for thiophanate-methyl is the sum of thiophanate-methyl and carbendazim, expressed as carbendazim (https://www.fao.org/agriculture/crops/thematic-sitemap/theme/pests/lpe/lpe-t/en/). On this basis, we conducted independent risk assessments for thiamethoxam and clothianidin. For thiophanate-methyl, measured residue concentrations were converted into carbendazim equivalents when calculating dietary exposure risks, with reference to the ADI of carbendazim.

### 2.7 Data analysis

The ME, which reflects signal suppression or enhancement due to the cherry matrix, was quantified using Eq. (5):

(5) $$\mathrm{ME}\;\left(\%\right)=\left[\text{slope}\;\left(\text{matrix}\right)/\text{slope}\;\left(\text{solvent}\right)-1\right]\times 100$$

where slope (matrix) and slope (solvent) represent the slopes of the matrix-matched and solvent-based standard calibration curves, respectively.

The dissipation kinetics of pesticides in cherries were evaluated using a first-order kinetic model, as described by Eqs. (6) and (7):

(6) $$C_{t}=C_{0}\times e^{-}\mathrm{ᵏᵗ}$$

(7) $$t₁/₂=\ln\left(2\right)/k$$

where C_0_ and C_t_ (μg/kg) refer to the initial residue concentration and the residue concentration at time t (days), respectively; k denotes the dissipation rate constant; and t₁/₂ (days) represents the half-life.

The PF was calculated to evaluate changes in pesticide residue levels following the processing of cherries, according to Eq. (8):

(8) $$\mathrm{PF}=C_{\mathrm{pc}}/C_{\mathrm{rac}}$$

where C_pc_ and C_rac_ correspond to the residue concentrations in the processed commodity and the raw agricultural commodity, respectively. A PF greater than 1 indicates concentration of the pesticide during processing, while a PF less than 1 indicates a reduction in residue levels.

## 3 Results

### 3.1 Method validation

The method validation data are summarized in Table 1. Both solvent-based and matrix-matched calibration curves demonstrated satisfactory linearity over the concentration range of 1–1000 μg/kg, with correlation coefficients (R^2^) ranging from 0.9909 to 0.9999. The LOQs were 1 μg/kg for cherries and 5 μg/kg for dried cherries for all analytes. The ME varied widely among analytes, ranging from −77.87% to 98.78%. To compensate for these substantial matrix effects, matrix-matched calibration standards were used for quantification of all target compounds. Method accuracy and precision were evaluated through recovery experiments and relative standard deviations (RSDs). The average recoveries for the pesticides ranged from 72.45% to 112.25%, with RSDs between 0.58% and 11.43%. These results fully satisfy the accepted validation criteria (recoveries of 70–120% and RSDs ≤20%). In conclusion, the validated method is suitable for the simultaneous determination of the target analytes in cherries.

**Table 1:** Regression equations, correlation coefficient (R^2^), matrix effect (ME), average recoveries (%) and relative standard deviation (RSD, %) for different pesticides.

Pesticides	Matrix	Regression equations	R^2^	ME (%)	Average recovery±RSD(%)		
					1 or 5 μg/kg	100 μg/kg	2000 μg/kg
Acetamiprid	acetonitrile	y = 13,399× + 159,335	0.9947	–	–	–	–
	cherries	y = 12,003× + 321,330	0.9957	−10.42	80.73 ± 3.80	84.36 ± 1.00	82.05 ± 2.58
	dried cherries	y = 9345× + 259,699	0.9944	−30.26	74.43 ± 3.49	76.06 ± 1.38	77.18 ± 1.91
Acetamiprid-*N*-desmethyl	acetonitrile	y = 23,353× + 60,279	0.9950	–	–	–	–
	cherries	y = 14,781× + 38,288	0.9985	−36.71	87.88 ± 3.65	91.52 ± 2.21	89.17 ± 0.89
	dried cherries	y = 12,085× + 29,023	0.9983	−48.25	83.95 ± 3.05	85.91 ± 2.66	86.66 ± 1.63
Carbendazim	acetonitrile	y = 190,336× + 442,503	0.9965	–	–	–	–
	cherries	y = 53,278× + 39,347	0.9992	−72.01	83.63 ± 4.65	88.10 ± 3.12	85.02 ± 0.58
	dried cherries	y = 42,115× + 31,510	0.9989	−77.87	74.31 ± 4.66	77.60 ± 3.48	76.94 ± 1.54
Clothianidin	acetonitrile	y = 4186× + 13,524	0.9921	–	–	–	–
	cherries	y = 2800× + 2380	0.9992	−33.11	74.97 ± 5.17	78.15 ± 2.54	75.50 ± 2.34
	dried cherries	y = 1936× + 2041	0.9988	−53.75	80.55 ± 3.27	81.01 ± 2.37	82.17 ± 1.35
Difenoconazole	acetonitrile	y = 54,323× + 26,367	0.9993	–	–	–	–
	cherries	y = 51,043× + 150,656	0.9988	−6.04	76.88 ± 4.00	80.05 ± 2.65	79.41 ± 1.63
	dried cherries	y = 40,516× - 91,601	0.9999	−25.42	74.29 ± 1.59	74.14 ± 2.39	75.54 ± 4.48
Fluopyram	acetonitrile	y = 27,982× + 262,281	0.9963	–	–	–	–
	cherries	y = 25,037× + 230,449	0.9971	−10.52	78.67 ± 3.68	82.84 ± 1.76	84.14 ± 2.76
	dried cherries	y = 15,750× + 210,893	0.9937	−43.71	77.05 ± 0.68	78.27 ± 2.74	76.18 ± 5.54
Imidacloprid	acetonitrile	y = 3089× + 35,182	0.9954	–	–	–	–
	cherries	y = 2675× + 28,175	0.9957	−13.40	81.66 ± 4.08	85.06 ± 1.36	81.50 ± 1.41
	dried cherries	y = 1916× + 20,419	0.9947	−37.97	73.24 ± 2.92	74.17 ± 2.51	74.38 ± 1.54
Kresoxim-methyl	acetonitrile	y = 5402× + 32	0.9976	–	–	–	–
	cherries	y = 4603× + 14,755	0.9970	−14.79	90.66 ± 4.16	94.27 ± 4.28	94.20 ± 1.25
	dried cherries	y = 3160× + 14,528	0.9961	−41.50	77.40 ± 2.43	76.36 ± 3.27	82.42 ± 7.89
Mefentrifluconazole	acetonitrile	y = 44,163× - 60,142	0.9999	–	–	–	–
	cherries	y = 42,140× + 12,568	0.9999	−4.58	72.45 ± 3.02	74.99 ± 2.80	75.00 ± 1.99
	dried cherries	y = 32,647× - 99,425	0.9997	−26.08	75.09 ± 1.42	75.07 ± 2.56	77.17 ± 4.79
Prochloraz	acetonitrile	y = 40,183× + 477,602	0.9954	–	–	–	–
	cherries	y = 38,241× + 259,854	0.9982	−4.83	78.65 ± 3.42	83.59 ± 1.8	84.28 ± 1.5
	dried cherries	y = 27,154× + 212,832	0.9978	−32.42	73.32 ± 2.83	73.72 ± 2.54	77.39 ± 5.58
Pyraclostrobin	acetonitrile	y = 30,709× + 70,913	0.9975	–	–	–	–
	cherries	y = 31,752× + 169,101	0.9933	3.40	77.43 ± 5.13	83.42 ± 4.98	85.27 ± 1.22
	dried cherries	y = 18,666× + 116,439	0.9909	−39.22	78.51 ± 2.26	78.19 ± 1.83	87.24 ± 9.65
Pyridaben	acetonitrile	y = 27,862× + 52,794	0.9967	–	–	–	–
	cherries	y = 37,129× + 239,370	0.9968	33.26	99.84 ± 4.65	110.51 ± 4.39	99.34 ± 2.37
	dried cherries	y = 55,385× + 253,879	0.9991	98.78	102.82 ± 3.92	110.23 ± 1.54	112.25 ± 2.03
Spirodiclofen	acetonitrile	y = 4449× - 18,189	0.9994	–	–	–	–
	cherries	y = 4802× + 16,026	0.9991	7.93	79.12 ± 3.56	85.09 ± 4.05	89.87 ± 1.95
	dried cherries	y = 3271× + 7830	0.9997	−26.48	76.80 ± 1.21	77.77 ± 2.25	87.98 ± 11.43
Tebuconazole	acetonitrile	y = 51,740× + 291,185	0.9969	–	–	–	–
	cherries	y = 26,880× + 218,897	0.9932	−48.05	90.07 ± 1.05	91.20 ± 2.39	93.17 ± 1.60
	dried cherries	y = 25,139× + 679,233	0.9995	−51.41	82.71 ± 2.63	81.22 ± 2.31	84.72 ± 2.42
Thiamethoxam	acetonitrile	y = 6655× + 101,344	0.9918	–	–	–	–
	cherries	y = 4989× + 49,806	0.9963	−25.03	82.96 ± 5.95	88.68 ± 5.11	85.53 ± 1.77
	dried cherries	y = 3744× + 39,987	0.9951	−43.74	76.92 ± 6.17	79.04 ± 4.12	79.47 ± 3.17
Thiophanate-methyl	acetonitrile	y = 18,357× + 60,423	0.9989	–	–	–	–
	cherries	y = 15,416× + 69,241	0.9942	−16.02	78.04 ± 9.20	87.19 ± 2.65	82.74 ± 7.52
	dried cherries	y = 10,565× + 46,598	0.9938	−42.45	75.62 ± 6.72	77.01 ± 2.16	82.16 ± 3.29
Trifloxystrobin	acetonitrile	y = 48,031× + 236,131	0.9970	–	–	–	–
	cherries	y = 43,390× + 388,070	0.9958	−9.66	79.41 ± 6.13	85.86 ± 2.11	86.03 ± 2.78
	dried cherries	y = 35,381× + 353,095	0.9957	−26.34	83.83 ± 5.52	87.03 ± 2.63	89.12 ± 3.39

### 3.2 Dissipation and metabolism of pesticides in cherries in the field

The dissipation behavior of pesticide residues in cherries under field conditions is summarized in Table 2 and Fig. 1. For the 14 parent pesticides, the initial concentrations (measured 2 h after application) ranged from 195.94 to 1465.58 μg/kg. These concentrations progressively declined over the sampling period. By the end of the experiment (14 days after application), the degradation rates of the 14 pesticides varied between 36.01% and 92.35% (Table S5), reflecting differences in their degradation potential. The dissipation in pesticide concentrations followed first-order kinetics, with R^2^ ranging from 0.8482 to 0.9694. The half-lives of these pesticides in cherries were calculated to be between 3.11 and 21.66 days. Fluopyram, pyraclostrobin and difenoconazole exhibited the highest residual persistence among the pesticides studied. The metabolite acetamiprid-*N*-desmethyl (derived from acetamiprid) was not detected in any field cherry samples. In contrast, carbendazim and clothianidin (metabolites of thiophanate-methyl and thiamethoxam, respectively) were detected in all samples, with concentration ranges of 160.50–443.05 μg/kg and 2.24–52.80 μg/kg, respectively. Additionally, the residual levels of these two metabolites initially increased, reaching peak values before gradually declining throughout the sampling period.

**Table 2:** Initial deposition, dynamic equation, correlation coefficient (R^2^) and half-lives (t₁/₂) of different pesticides in cherries in the field.

Pesticides	Initial deposition (μg/kg)	Dynamic equation	R^2^	t₁/₂ (d)
Acetamiprid	235.40 ± 2.85	y = 256.70e^-0.079x^	0.9545	8.77
Difenoconazole	1139.52 ± 23.66	y = 1117.09e^-0.037x^	0.8555	18.73
Fluopyram	1043.37 ± 17.59	y = 1063.51e^-0.032x^	0.9529	21.66
Imidacloprid	195.94 ± 2.05	y = 211.98e^-0.071x^	0.9076	9.76
Kresoxim-methyl	540.57 ± 21.93	y = 523.16e^-0.048x^	0.9588	14.44
Mefentrifluconazole	803.49 ± 6.39	y = 739.92e^-0.043x^	0.8482	16.12
Prochloraz	688.00 ± 52.54	y = 707.80e^-0.193x^	0.9380	3.59
Pyraclostrobin	595.63 ± 19.17	y = 539.97e^-0.035x^	0.8654	19.80
Pyridaben	881.24 ± 27.02	y = 874.30e^-0.223x^	0.8579	3.11
Spirodiclofen	283.89 ± 10.42	y = 309.17e^-0.083x^	0.9521	8.35
Tebuconazole	451.04 ± 4.03	y = 482.22e^-0.065x^	0.9145	10.66
Thiamethoxam	754.72 ± 12.58	y = 795.15e^-0.070x^	0.9110	9.90
Thiophanate-methyl	1465.58 ± 9.13	y = 1372.00e^-0.050x^	0.9694	13.86
Trifloxystrobin	649.73 ± 8.74	y = 770.27e^-0.072x^	0.8503	9.63

**Fig. 1:**
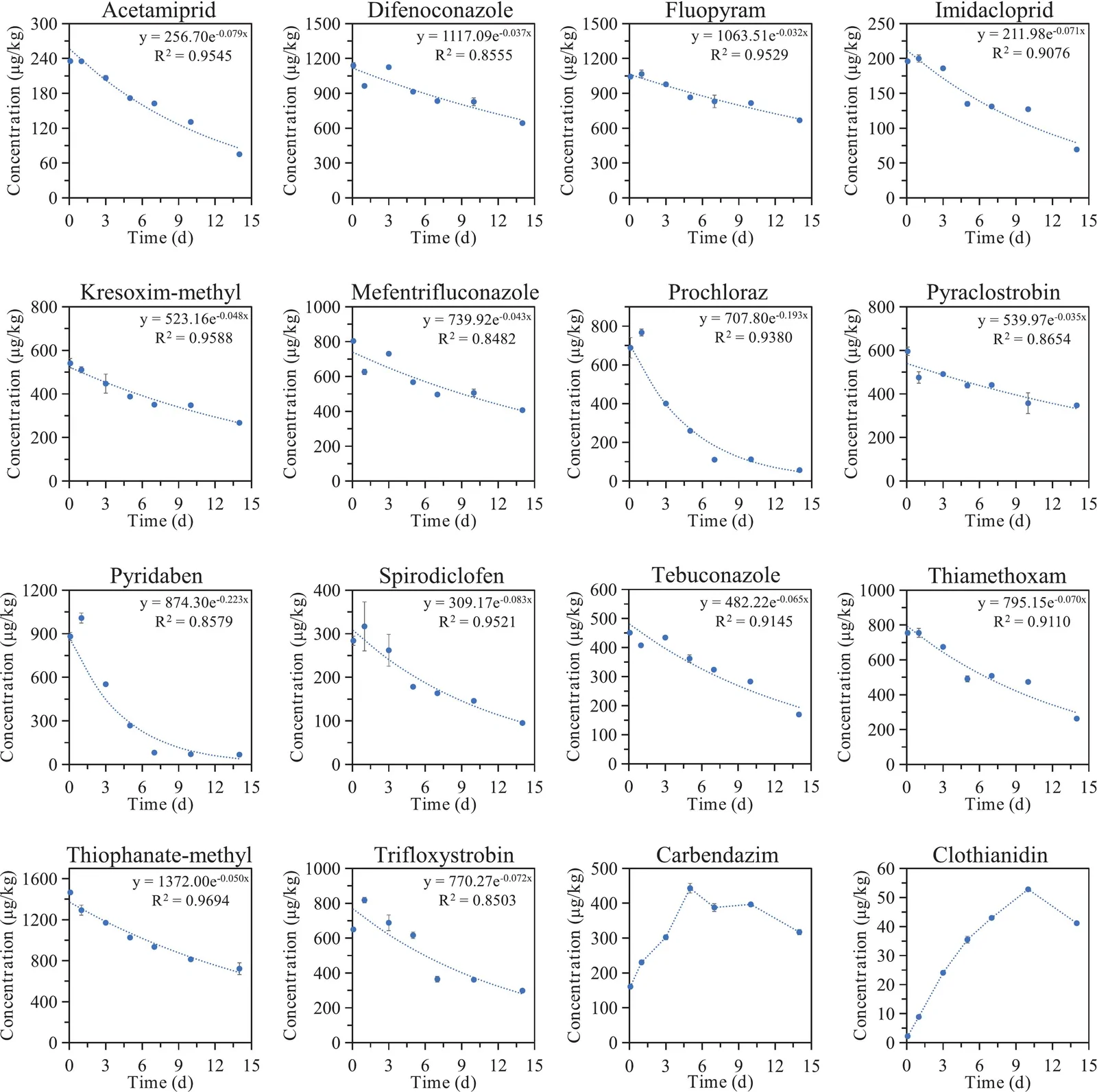
The dissipation curves of different pesticides in cherries.

### 3.3 Residue changes of pesticides during cherry processing

This study investigated the residue changes of 14 parent pesticides and 2 metabolites (carbendazim and clothianidin) during cherry processing, including washing and the production of dried cherries via oven drying and microwave drying. The results are summarized in Fig. 2 and Table S6. During the washing treatment, varying degrees of pesticide residue removal were observed depending on washing duration (1 to 10 min), with PFs ranging from 0.10 to 0.98. An exception was noted for the metabolite clothianidin at 1, 2 and 5 min of washing, where PFs ranged from 1.06 to 1.23 (Fig. 2A). Furthermore, a general trend was identified whereby longer washing time corresponded to improved pesticide removal, indicating a negative correlation between residual pesticide levels and washing duration. In the drying treatment, PFs for oven-dried cherries ranged from 2.99 to 6.70, while those for microwave-dried cherries ranged from 0.75 to 3.93. These results suggest that the drying process generally led to the concentration of pesticide residues in the final dried product. Additionally, oven drying was found to yield higher residual pesticide concentrations compared to microwave drying (Fig. 2B).

**Fig. 2:**
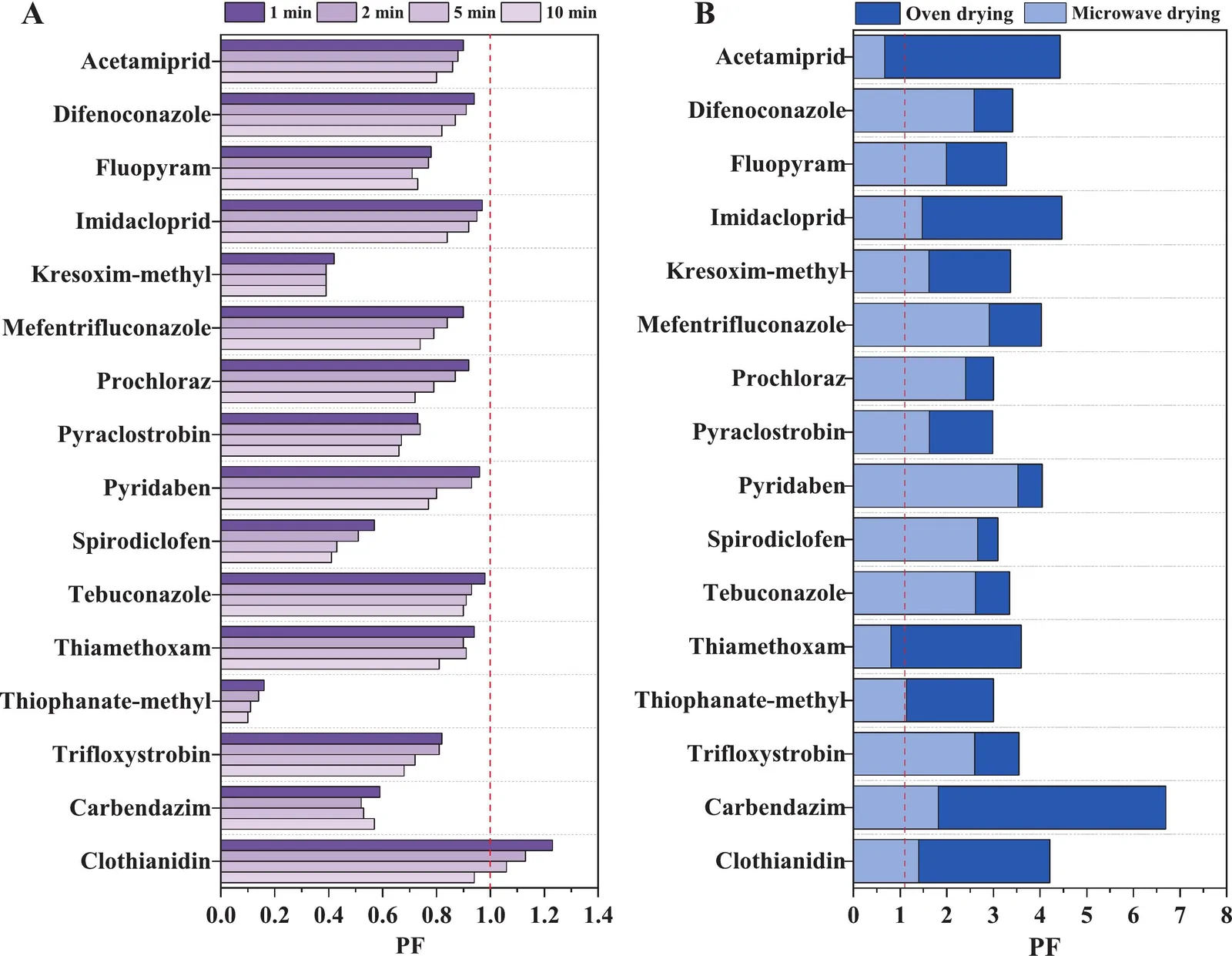
The processing factors (PFs) of different pesticides in cherries during washing (A) and drying (B).

### 3.4 Dietary risk assessment of pesticides in cherries

Chronic and acute dietary risks were assessed based on pesticide residue levels in cherries sampled at different intervals and on cherry consumption data for children and adults. The results of the dietary risk assessment for various pesticides are summarized in Fig. 3 and Table S7. For chronic dietary risk, the calculated RQc values for children and adults were 5.52 × 10^−8^ − 2.18 × 10^−4^ and 1.33 × 10^−8^ − 6.75 × 10^−5^ for intervals of 2 h, 2.19 × 10^−7^ − 2.63 × 10^−4^ and 5.26 × 10^−8^ − 6.32 × 10^−5^ for 1 d, 5.94 × 10^−7^ − 2.78 × 10^−4^ and 1.43 × 10^−7^ − 6.67 × 10^−5^ for 3 d, 8.76 × 10^−7^ − 2.26 × 10^−4^ and 2.10 × 10^−7^ − 5.42 × 10^−5^ for 5 d, 1.06 × 10^−6^ − 2.06 × 10^−4^ and 2.55 × 10^−7^ − 4.94 × 10^−5^ for 7 d, 1.30 × 10^−6^ − 2.04 × 10^−4^ and 3.13 × 10^−7^ − 4.90 × 10^−5^ for 10 d and 1.02 × 10^−6^ − 1.65 × 10^−4^ and 2.44 × 10^−7^ − 3.96 × 10^−5^ for 14 d, respectively, which were substantially below 1 (Fig. 3A, B). Among the pesticides evaluated, difenoconazole and fluopyram exhibited relatively higher RQc values. In the acute dietary risk assessment, kresoxim-methyl, spirodiclofen and trifloxystrobin were excluded due to the absence of established ARfD. For the remaining pesticides, the RQa values for children and adults were 4.31 × 10^−5^ − 0.204 and 4.09 × 10^−5^ − 0.193 for 2 h, 1.71 × 10^−4^ − 0.233 and 1.62 × 10^−4^ − 0.221 for 1 d, 4.64 × 10^−4^ − 0.128 and 4.40 × 10^−4^ − 0.121 for 3 d, 6.84 × 10^−4^ − 0.118 and 6.49 × 10^−4^ − 0.111 for 5 d, 8.29 × 10^−4^ − 0.105 and 7.86 × 10^−4^ − 0.100 for 7 d, 1.02 × 10^−3^ − 0.099 and 9.65 × 10^−4^ − 0.093 for 10 d and 7.94 × 10^−4^ − 0.083 and 7.52 × 10^−4^ − 0.079 for 14 d, respectively, which were also below 1 (Fig. 3C, D). Notably, pyridaben, pyraclostrobin and thiophanate-methyl showed higher RQa values compared to other pesticides. In summary, both chronic and acute dietary risks associated with pesticide exposure from cherry consumption were found to be within acceptable limits.

**Fig. 3:**
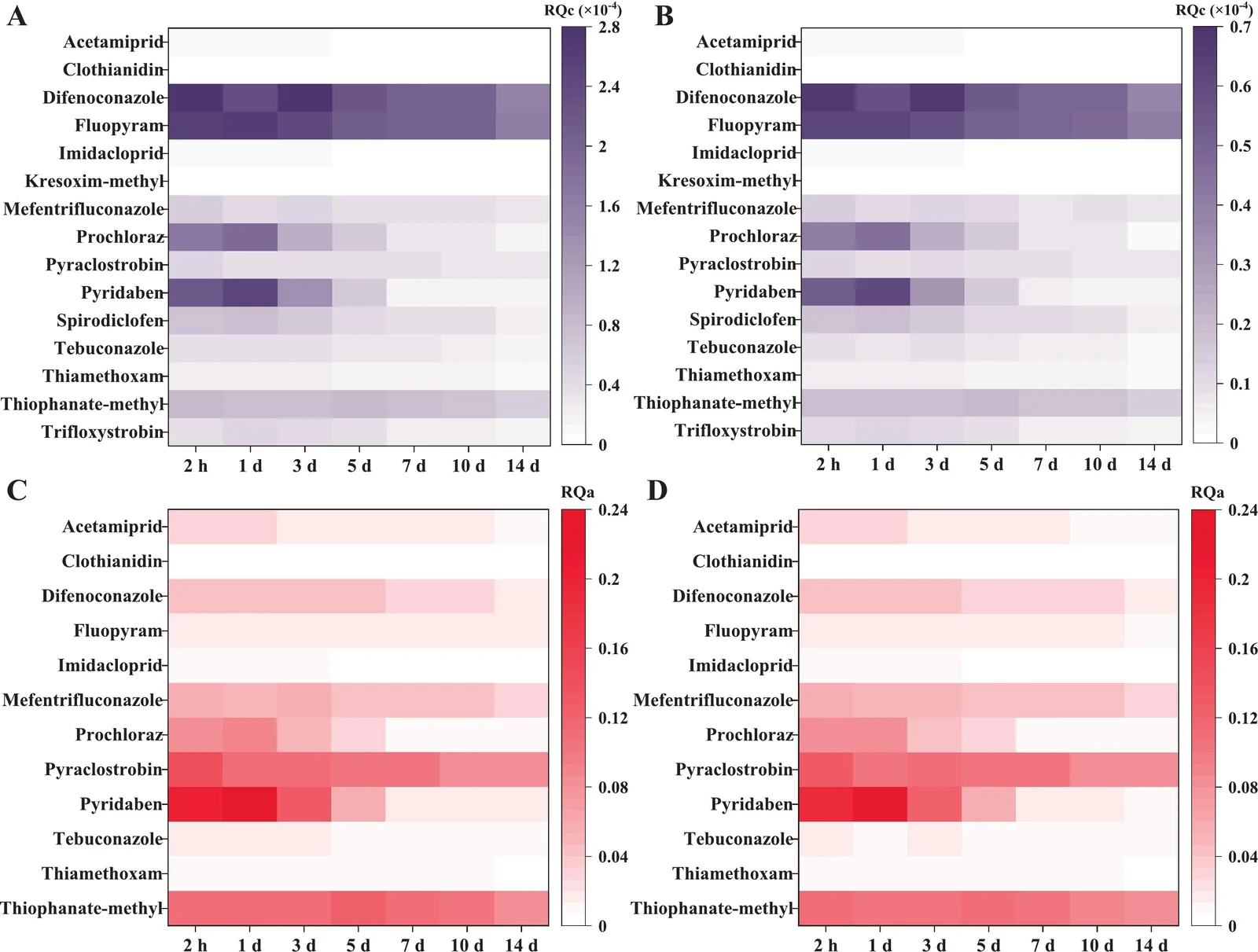
Heatmaps of the chronic and acute risk quotient (RQc and RQa, respectively) values of different pesticides for children (A, C) and adults (B, D) from cherry consumption.

## 4 Discussion

Understanding the dissipation kinetics and half-lives of pesticides is critical for assessing their persistence and predicting residue dynamics in crops, thereby guiding rational pesticide application and ensuring food safety (Cui et al., 2025). The current study reveals a considerable variation in pesticide half-lives in cherries, ranging from 3.11 to 21.66 days. Notably, fluopyram, pyraclostrobin, difenoconazole and mefentrifluconazole exhibited high persistence and slow dissipation, with half-lives exceeding 16 days (Li et al., 2021). Limited data are available on the dissipation patterns of pesticide residues in cherries. Balkan et al. (2025) reported acetamiprid half-lives of 4.41–12.6 days in cherries, whether applied individually or in mixtures, which aligns with our finding of 8.77 days. In contrast, the reported half-lives for pyraclostrobin (3.43–10.50 days) were shorter than the value observed in our study (19.80 days). Moreover, Yao et al. (2021) documented a fludioxonil half-life of 33.7–44.7 days in cherries, which exceed those of all pesticides tested in our work. Multiple factors influence pesticide dissipation in cherries, including physicochemical properties of the pesticides, application rates and time, environmental conditions (e.g., temperature, moisture and solar radiation) and crop variety (Cui et al., 2025; Li, Xiao, & Zhu, 2024). Furthermore, the growth-related increase in fruit weight and surface area may contribute to a dilution effect, thereby reducing residue concentrations (Cui et al., 2023; Noh et al., 2019). In this study, metabolites such as carbendazim and clothianidin were detected in cherries at concentrations ranging from 2.24 to 443.05 μg/kg. These compounds are also widely used as active ingredients in commercial pesticide formulations. Their frequent detection as metabolites in various crops has been corroborated by previous studies (Bian et al., 2023; Liu et al., 2021; Rasool et al., 2024; Wang et al., 2025). Due to their inherent toxicity and potential interactive effects with parent pesticides, continuous monitoring and risk assessment of these metabolites are warranted.

Our study demonstrated that only 36.01–92.35% of pesticide residues had degraded by 14 days after application, indicating that a considerable portion of residues persisted in cherries. As cherries are typically consumed fresh and are commonly washed before eating, we evaluated the effect of washing on residue removal. The results revealed that washing eliminated varying amounts of pesticide residues, with a negative correlation observed between residual levels and washing duration. These findings are consistent with previous studies confirming that washing is a technically simple, rapid, and cost-effective method for substantially reducing pesticide residues in various foods (Acoglu & Omeroglu, 2021; Carreiró et al., 2025; Gao et al., 2024; Sivasankari et al., 2025). Notably, certain metabolites, such as clothianidin, exhibited a slight increase in residue levels after washing, potentially due to the hydrolysis of thiamethoxam during the process. In recent years, cherry drying has gained popularity as a processing method to concentrate nutritional content and extend shelf life, resulting in products commonly consumed as snacks. Our results indicated that pesticide residues, including detected metabolites, became concentrated during drying, with maximum concentration factors of 6.70 for oven drying and 3.93 for microwave drying. The net residue level in processed samples is determined by the balance between water sublimation (which tends to concentrate residues) and pesticide degradation or evaporation (which reduces them), as described in previous study (Cui et al., 2025). Similar concentration effects have been reported in various dried fruits, including raisins, apples, peaches and bananas (Cui et al., 2025; Hrynko et al., 2023; Liu et al., 2023; Ma et al., 2025). Moreover, oven drying resulted in higher residual pesticide concentrations compared to microwave drying. We hypothesize that drying temperature is a key factor influencing this difference. While oven drying was conducted at 60 °C, microwave drying involves substantially higher temperatures. The elevated temperatures in microwave drying likely enhance pesticide degradation, leading to lower residual levels. This aligns with the general understanding that higher drying temperatures promote pesticide degradation in processed products (Bian et al., 2023). Beyond thermal degradation, microwave drying may remove pesticides via vapor-stripping, porous tissue formation and short-term high-intensity heating, while oven drying lacks such effects, compacts cells and concentrates residues with limited pesticide degradation (Zhang et al., 2022).

The results of both chronic and acute dietary risk assessments demonstrated that the evaluated pesticide residues in cherries fell within acceptable limits for children and adults, as indicated by RQ values below 1. This finding is consistent with several studies reporting no significant health risks associated with dietary pesticide exposure from cherry consumption based on field data (Balkan et al., 2025; Yao et al., 2021). Among the pesticides assessed, difenoconazole and fluopyram were associated with relatively higher chronic risk, whereas pyridaben, pyraclostrobin and thiophanate-methyl exhibited greater acute dietary risks. These observations are likely attributable to their elevated residue concentrations in cherries and their comparatively lower ADI or ARfD values (Table S4). Despite the acceptable RQ values, the potential health risks arising from pesticide exposure via cherry consumption should not be overlooked. First, multiple pesticides are frequently applied simultaneously or sequentially in cherry cultivation, and the co-occurrence of pesticide residues may lead to cumulative toxic effects in humans (Caldas, 2023; de Mendonça Pereira & Spisso, 2026; Yang et al., 2024). Furthermore, under the influence of biological or abiotic factors, certain pesticides in cherries may give rise to toxic metabolites in humans, which could act synergistically with the parent compounds. Second, in addition to cherry consumption, humans can be exposed to pesticides through multiple other pathways. Pesticide residues can be detected in a variety of other dietary sources including fruits, vegetables, cereals and seafood, which ultimately gives rise to cumulative exposure risk (Sim et al., 2026; Zhou et al., 2023). Third, pesticide residue levels may increase during post-harvest processing—such as during drying—thereby elevating dietary exposure risks. Fourth, although low-dose exposure to certain pesticides may not elicit immediate toxic symptoms, multi-omics studies—including metabolomics, transcriptomics, genomics and proteomics—have revealed that even permitted levels of pesticide exposure can induce functional alterations in mammals (Mesnage et al., 2021; Wen et al., 2024; Zhang et al., 2026).

We acknowledge that the current study has several limitations. First, only washing and drying methods were included in the processing of cherries. In practice, additional techniques such as producing cherry sauce and juice should be incorporated to fully assess changes in pesticide residue levels. Second, the drying method implemented reflects household practices and may differ to some extent from industrial-scale processing. Third, the analysis was confined to several major metabolites, whereas further toxicologically relevant metabolites should be included in future monitoring efforts. Fourth, the field experiment of this study was conducted at only one experimental site in Jinan, Shandong Province. Therefore, the extrapolation of the obtained results to other environmental and agronomic conditions may be limited. To further verify the conclusions drawn from this study, multi-location field trials are needed for future research.

## 5 Conclusions

To our knowledge, this study represents the first comprehensive investigation into the dissipation, metabolism, PFs, and risk assessment of 14 commonly used pesticides in cherries throughout the field-to-processing continuum. Field trial results indicated that the dissipation of all tested pesticides followed first-order kinetics, with half-lives ranging from 3.11 to 21.66 days. Additionally, the metabolites carbendazim and clothianidin were detected in field-grown cherries at concentrations between 2.24 and 443.05 μg/kg. Washing treatments effectively removed varying amounts of pesticide residues, and a negative correlation was observed between residue levels and washing duration. In contrast, pesticide residues became concentrated during drying, with maximum PFs reaching 6.70 for oven drying and 3.93 for microwave drying. Moreover, oven drying resulted in higher residual pesticide concentrations compared to microwave drying. Both chronic and acute dietary risk assessments indicated that health risks associated with the consumption of cherries containing the studied pesticide residues were within acceptable limits. Accordingly, these 14 pesticides are recommended for field use by farmers following the doses and application frequencies adopted in the present study. Furthermore, microwave drying is preferable for household-scale preparation of dried cherry products to reduce potential pesticide-residue risks. Appropriately prolonging the washing duration of cherries can effectively facilitate the removal of pesticide residues. Overall, this study elucidates the dissipation behavior of multiple pesticides in cherries under field conditions and documents residue changes during processing, thereby providing a scientific basis for the rational application of pesticides and the promotion of food safety in cherry consumption.

## CRediT authorship contribution statement

**Kai Cui:** Writing – review & editing, Writing – original draft, Methodology, Funding acquisition, Formal analysis, Data curation. **Liping Fang:** Resources, Methodology. **Ruiyan Ding:** Methodology, Investigation. **Wanru Su:** Resources. **Qiang Liu:** Supervision. **Shuai Guan:** Validation. **Jingyun Liang:** Software. **Jian Wang:** Supervision. **Teng Li:** Visualization. **Zhan Dong:** Writing – review & editing, Project administration, Funding acquisition, Conceptualization.

## Declaration of competing interest

The authors declare that they have no known competing financial interests or personal relationships that could have appeared to influence the work reported in this paper.

## Data Availability

No data was used for the research described in the article.
