# Supplementary material for: From orchard to table: Insight into the dissipation, metabolism, processing and risk assessment of 14 pesticides in sweet cherry
Source: Food Chem X. 2026 Oct 19;40:104659. doi: 10.1016/j.fochx.2026.104659 (PMC13717002; doi:10.1016/j.fochx.2026.104659)
Supplement: Supplementary file 1 [file mmc1.docx]

**From orchard to table: Insight into the dissipation, metabolism, processing and risk assessment of 14 pesticides in sweet cherry**

Kai Cui^a^, Liping Fang^a^, Ruiyan Ding^a^, Wanru Su^a^**^,^** ^b^, Qiang Liu^c^, Shuai Guan^a^, Jingyun Liang^a^, Jian Wang^a^, Teng Li^a^, Zhan Dong^a^**^,^ ***

^a^ Institute of Agricultural Products Quality Safety and Standards, Shandong Academy of Agricultural Sciences; Shandong Provincial Key Laboratory of Test Technology on Food Quality and Safety, Jinan, Shandong, 250100, People’s Republic of China

^b^ College of Plant Protection, Shandong Agricultural University, Tai’an, Shandong 271018, People’s Republic of China

^c^ Lunan Pharmaceutical Group Co. Ltd., Linyi, Shandong, 276006, People’s Republic of China

*** Corresponding author:**

E-mail: [zhandongsaas@163.com](mailto:zhandongsaas@163.com) (Zhan Dong)

**Table captions**

**Table S1.** The physicochemical properties of 16 pesticides.

**Table S2.** Application parameters of 12 commercial pesticide products on cherries.

**Table S3.** Optimized HPLC–MS/MS parameters of different pesticides.

**Table S4.** Acceptable daily intake (ADI) and acute reference dose (ARfD) of different pesticides.

**Table S5.** Residue levels and degradation rates of different pesticides in cherries.

**Table S6.** Residue levels and processing factors (PFs) of different pesticides during cherry processing.

**Table S7.** The estimated daily intake (EDI), estimation of short term intake (ESTI), chronic risk quotient (RQc) and acute risk quotient (RQa) values of different pesticides for children and the adults from cherry consumption.

**Table S1.** The physicochemical properties of 16 pesticides.

| Pesticide | Chemical formula | Molecular mass | Solubility in water at 20 °C at pH 7 (mg/L) | Melting point (°C) | Degradation point (°C) | Octanol-water partition coefficient at pH 7, 20 °C | Vapour pressure at 20 °C (mPa) |
| --- | --- | --- | --- | --- | --- | --- | --- |
| Acetamiprid | C₁₀H₁₁ClN₄ | 222.67 | 2950 | 98.9 | 200 | 0.8 | 0.001 |
| Difenoconazole | C₁₉H₁₇Cl₂N₃O₃ | 406.26 | 15 | 82.5 | 337 | 4.36 | 0.0000333 |
| Fluopyram | C₁₆H₁₁ClF₆N₂O | 396.76 | 16 | 117.5 | 300 | 3.3 | 0.0012 |
| Imidacloprid | C₉H₁₀ClN₅O₂ | 255.66 | 610 | 144 | 230 | 0.57 | 0.0000004 |
| Kresoxim-methyl | C₁₈H₁₉NO₄ | 313.35 | 2 | 102 | 310 | 3.4 | 0.0023 |
| Mefentrifluconazole | C₁₈H₁₅ClF₃N₃O₂ | 397.78 | 0.81 | 126 | 300 | 3.4 | 0.0032 |
| Prochloraz | C₁₅H₁₆Cl₃N₃O₂ | 376.7 | 26.5 | 48.3 | 220 | 3.5 | 0.15 |
| Pyraclostrobin | C₁₉H₁₈ClN₃O₄ | 387.82 | 1.9 | 63.7 | 200 | 3.99 | 0.000026 |
| Pyridaben | C₁₉H₂₅ClN₂OS | 364.93 | 0.022 | 109.4 | 200 | 6.37 | 0.001 |
| Spirodiclofen | C₂₁H₂₄Cl₂O₄ | 411.32 | 0.05 | 94.8 | - | 5.1 | 0.0003 |
| Tebuconazole | C₁₆H₂₂ClN₃O | 307.82 | 36 | 105 | 350 | 3.7 | 0.0013 |
| Thiamethoxam | C₈H₁₀ClN₅O₃S | 291.71 | 4100 | 139.1 | 147 | -0.13 | 0.0000066 |
| Thiophanate-methyl | C₁₂H₁₄N₄O₄S₂ | 342.39 | 18.5 | - | 165 | 1.4 | 0.009 |
| Trifloxystrobin | C₂₀H₁₉F₃N₂O₄ | 408.37 | 0.61 | 72.9 | 285 | 4.5 | 0.0034 |
| Carbendazim | C₉H₉N₃O₂ | 191.21 | 8 | 305 | 305 | 1.48 | 0.09 |
| Clothianidin | C₆H₈ClN₅O₂S | 249.7 | 327 | 176.8 | - | 0.905 | 0.000000038 |

**Table S2.** Application parameters of 12 commercial pesticide products on cherries.

| Pesticides | Formulation | Contents | Application dosages/ dilution multiple | Application volume | Application times | Application interval (d) |
| --- | --- | --- | --- | --- | --- | --- |
| Acetamiprid | wettable powder | 20% | 6000 | 2 L/tree | 1 | - |
| Difenoconazole | water dispersible granule | 10% | 1000 | 2 L/tree | 2 | 7 |
| Fluopyram and Trifloxystrobin | suspension concentrate | 43% (21.5%+21.5%) | 1500 | 2 L/tree | 2 | 7 |
| Imidacloprid | wettable powder | 10% | 4000 | 2 L/tree | 1 | - |
| Kresoxim-methyl | water dispersible granule | 50% | 3000 | 2 L/tree | 2 | 7 |
| Mefentrifluconazole and Pyraclostrobin | suspension concentrate | 400 g/L (200 g/L+200 g/L) | 2700 | 2 L/tree | 3 | 7 |
| Prochloraz | emulsifiable concentrate | 25% | 2000 | 2 L/tree | 3 | 7 |
| Pyridaben | emulsifiable concentrate | 15% | 1500 | 2 L/tree | 1 | - |
| Spirodiclofen | suspension concentrate | 240 g/L | 4000 | 2 L/tree | 1 | - |
| Tebuconazole | suspension concentrate | 43% | 4000 | 2 L/tree | 2 | 7 |
| Thiamethoxam | suspension concentrate | 21% | 2000 | 2 L/tree | 1 | - |
| Thiophanate-methyl | wettable powder | 70% | 800 | 2 L/tree | 3 | 7 |

**Table S3.** Optimized HPLC–MS/MS parameters of different pesticides.

| Pesticides | Quantitative ion pair (m/z) | Qualitative ion pair (m/z) | Fragmentor  (V) | Collision Energy  (V) | Ion polarity |
| --- | --- | --- | --- | --- | --- |
| Acetamiprid | 223.08/126.1 | 223.08/73 | 166 | 20/72 | Positive |
| Difenoconazole | 406.07/251 | 406.07/337 |  | 28/16 | Positive |
| Fluopyram | 397/173 | 397/207.9 |  | 40/20 | Positive |
| Imidacloprid | 256.06/175.1 | 256.06/209 |  | 20/12 | Positive |
| Kresoxim-methyl | 314.1/267 | 314.1/222 |  | 3/8 | Positive |
| Mefentrifluconazole | 398.1/70 | 398.1/182.1 |  | 50/35 | Positive |
| Prochloraz | 376/307.9 | 376/265.8 |  | 5/15 | Positive |
| Pyraclostrobin | 388/163 | 388/194 |  | 20/20 | Positive |
| Pyridaben | 365.2/309 | 365.2/147 |  | 9/24 | Positive |
| Spirodiclofen | 411/71.1 | 411/313 |  | 15/10 | Positive |
| Tebuconazole | 308.1/70 | 308.1/150.9 |  | 20/30 | Positive |
| Thiamethoxam | 292.03/211 | 292.03/181 |  | 8/24 | Positive |
| Thiophanate-methyl | 343/150.7 | 343/311 |  | 12/9 | Positive |
| Trifloxystrobin | 409.14/186 | 409.14/145 |  | 20/60 | Positive |
| Metabolites |  |  |  |  |  |
| Acetamiprid-N-desmethyl | 209.06/126 | 209.06/90 |  | 16/36 | Positive |
| Carbendazim | 192.08/160 | 192.08/132 |  | 16/36 | Positive |
| Clothianidin | 250.02/169.1 | 250.02/132.1 |  | 12/16 | Positive |

**Table S4.** Acceptable daily intake (ADI) and acute reference dose (ARfD) of different pesticides.

| Pesticides | ADI  (μg/kg bw) | ARfD  (μg/kg bw) |
| --- | --- | --- |
| Acetamiprid | 70^a^ | 100^a^ |
| Difenoconazole | 10^a^ | 300^a^ |
| Fluopyram | 10^a^ | 500^a^ |
| Imidacloprid | 60^a^ | 400^a^ |
| Kresoxim-methyl | 400^a^ | unnecessary^a^ |
| Mefentrifluconazole | 35^b^ | 150^b^ |
| Prochloraz | 10^a^ | 100^a^ |
| Pyraclostrobin | 30^a^ | 50^a^ |
| Pyridaben | 10^b^ | 50^b^ |
| Spirodiclofen | 10^a^ | unnecessary^a^ |
| Tebuconazole | 30^a^ | 300^a^ |
| Thiamethoxam | 80^a^ | 1000^a^ |
| Thiophanate-methyl | 90^a^ | 1000^a^ |
| Trifloxystrobin | 40^a^ | unnecessary^a^ |
| Carbendazim | 30^a^ | 100^a^ |
| Clothianidin | 100^a^ | 600^a^ |

^a^ ADI or ARfD were obtained from the Joint FAO/WHO Meeting on Pesticide Residues (JMPR).

^b^ ADI or ARfD were obtained from the European Food Safety Authority (EFSA).

**Table S5.** Residue levels and degradation rates of different pesticides in cherries.

| Pesticides | Time | Residues (μg/kg) | Degradation rates (%) |
| --- | --- | --- | --- |
| Acetamiprid | 2 h | 235.4±2.85 | - |
|  | 1 d | 235.1±2.68 | 0.13 |
|  | 3 d | 206.45±3.86 | 12.30 |
|  | 5 d | 171.7±1.9 | 27.06 |
|  | 7 d | 162.51±2.04 | 30.97 |
|  | 10 d | 130.7±1.24 | 44.48 |
|  | 14 d | 75.08±0.76 | 68.11 |
| Difenoconazole | 2 h | 1139.52±23.66 | - |
|  | 1 d | 963.45±15.7 | 15.45 |
|  | 3 d | 1124.87±3.9 | 1.29 |
|  | 5 d | 913.79±2.38 | 19.81 |
|  | 7 d | 833.48±7.54 | 26.86 |
|  | 10 d | 827.13±32.9 | 27.41 |
|  | 14 d | 643.39±4.82 | 43.54 |
| Fluopyram | 2 h | 1043.37±17.59 | - |
|  | 1 d | 1066.36±33.82 | - |
|  | 3 d | 977.48±11.08 | 6.32 |
|  | 5 d | 865.3±4.06 | 17.07 |
|  | 7 d | 830.29±54.33 | 20.42 |
|  | 10 d | 815.97±6.29 | 21.79 |
|  | 14 d | 667.64±5.93 | 36.01 |
| Imidacloprid | 2 h | 195.94±2.05 | - |
|  | 1 d | 199.83±5.25 | - |
|  | 3 d | 185.83±2.08 | 5.16 |
|  | 5 d | 134.82±3.11 | 31.19 |
|  | 7 d | 131.13±2.51 | 33.08 |
|  | 10 d | 127.15±2.16 | 35.11 |
|  | 14 d | 69.39±0.57 | 64.59 |
| Kresoxim-methyl | 2 h | 540.57±21.93 | - |
|  | 1 d | 510.73±14.32 | 5.52 |
|  | 3 d | 447.23±43.7 | 17.27 |
|  | 5 d | 387.71±5.8 | 28.28 |
|  | 7 d | 350.59±5.33 | 35.14 |
|  | 10 d | 347.93±4.95 | 35.64 |
|  | 14 d | 267.23±1.81 | 50.57 |
| Mefentrifluconazole | 2 h | 803.49±6.39 | - |
|  | 1 d | 626.4±15.31 | 22.04 |
|  | 3 d | 730.44±4.13 | 9.09 |
|  | 5 d | 567.39±3.26 | 29.39 |
|  | 7 d | 496.6±2.51 | 38.19 |
|  | 10 d | 506.04±21.46 | 37.02 |
|  | 14 d | 406.9±5.75 | 49.36 |
| Prochloraz | 2 h | 688±52.54 | - |
|  | 1 d | 767.01±17.69 | - |
|  | 3 d | 400.31±6.01 | 41.82 |
|  | 5 d | 259.23±6.19 | 62.32 |
|  | 7 d | 110.2±1.53 | 83.98 |
|  | 10 d | 112.12±1.01 | 83.70 |
|  | 14 d | 56.37±0.75 | 91.81 |
| Pyraclostrobin | 2 h | 595.63±19.17 | - |
|  | 1 d | 475.26±26.01 | 20.21 |
|  | 3 d | 491.15±6.36 | 17.54 |
|  | 5 d | 438.6±11.27 | 26.36 |
|  | 7 d | 441±4.02 | 25.96 |
|  | 10 d | 357.26±47.26 | 40.02 |
|  | 14 d | 347.34±2.79 | 41.68 |
| Pyridaben | 2 h | 881.24±27.02 | - |
|  | 1 d | 1007.88±35.04 | - |
|  | 3 d | 552.18±2.03 | 37.34 |
|  | 5 d | 267.81±6.78 | 69.61 |
|  | 7 d | 81.26±1.62 | 90.78 |
|  | 10 d | 70.41±2.13 | 92.01 |
|  | 14 d | 67.39±1.19 | 92.35 |
| Spirodiclofen | 2 h | 283.89±10.42 | - |
|  | 1 d | 316.78±56.38 | - |
|  | 3 d | 261.97±36.48 | 7.72 |
|  | 5 d | 178.2±0.96 | 37.23 |
|  | 7 d | 163.41±1.04 | 42.44 |
|  | 10 d | 145.85±3.71 | 48.63 |
|  | 14 d | 95.08±1.82 | 66.51 |
| Tebuconazole | 2 h | 451.04±4.03 | - |
|  | 1 d | 407.73±4.76 | 9.60 |
|  | 3 d | 434.42±2.8 | 3.68 |
|  | 5 d | 362.07±12.66 | 19.73 |
|  | 7 d | 323.88±5.23 | 28.19 |
|  | 10 d | 283.11±0.67 | 37.23 |
|  | 14 d | 169.61±3.3 | 62.40 |
| Thiamethoxam | 2 h | 754.72±12.58 | - |
|  | 1 d | 754.78±25.72 | - |
|  | 3 d | 674.43±10.43 | 10.64 |
|  | 5 d | 490.64±16.44 | 34.99 |
|  | 7 d | 508.23±3.22 | 32.66 |
|  | 10 d | 473.53±7.16 | 37.26 |
|  | 14 d | 262.81±5.09 | 65.18 |
| Thiophanate-methyl | 2 h | 1465.58±9.13 | - |
|  | 1 d | 1292.44±49.3 | 11.81 |
|  | 3 d | 1169.78±10.92 | 20.18 |
|  | 5 d | 1025.77±10.55 | 30.01 |
|  | 7 d | 934.28±5.23 | 36.25 |
|  | 10 d | 813.1±8.04 | 44.52 |
|  | 14 d | 721.55±57.46 | 50.77 |
| Trifloxystrobin | 2 h | 649.73±8.74 | - |
|  | 1 d | 818.1±15.79 | - |
|  | 3 d | 688.15±44.77 | - |
|  | 5 d | 616.31±18.45 | 5.14 |
|  | 7 d | 364.56±15.7 | 43.89 |
|  | 10 d | 361.35±9.3 | 44.38 |
|  | 14 d | 298.76±10.05 | 54.02 |
| Carbendazim | 2 h | 160.5±2.33 | - |
|  | 1 d | 230.16±6.04 | - |
|  | 3 d | 302.31±6.85 | - |
|  | 5 d | 443.05±13.8 | - |
|  | 7 d | 387.72±11.03 | - |
|  | 10 d | 396.68±5.81 | - |
|  | 14 d | 317.13±7.46 | - |
| Clothianidin | 2 h | 2.24±0.08 | - |
|  | 1 d | 8.88±0.11 | - |
|  | 3 d | 24.06±0.65 | - |
|  | 5 d | 35.48±1.15 | - |
|  | 7 d | 42.97±0.23 | - |
|  | 10 d | 52.8±0.41 | - |
|  | 14 d | 41.15±0.63 | - |

**Table S6.** Residue levels and processing factors (PFs) of different pesticides during cherry processing.

| Pesticides | Processing | Residues (μg/kg) | PFs |
| --- | --- | --- | --- |
| Acetamiprid | raw cherries | 235.1±2.68 | - |
|  | washing (1 min) | 210.77±3.09 | 0.90 |
|  | washing (2 min) | 205.99±5.87 | 0.88 |
|  | washing (5 min) | 201.66±2.86 | 0.86 |
|  | washing (10 min) | 187.29±2.88 | 0.80 |
|  | oven drying | 1040.35±75.19 | 4.43 |
|  | microwave drying | 176.49±8.61 | 0.75 |
| Difenoconazole | raw cherries | 963.45±15.7 | - |
|  | washing (1 min) | 907.73±9.12 | 0.94 |
|  | washing (2 min) | 875.96±7.97 | 0.91 |
|  | washing (5 min) | 834.71±3.79 | 0.87 |
|  | washing (10 min) | 791.66±3.82 | 0.82 |
|  | oven drying | 3293.2±42.9 | 3.42 |
|  | microwave drying | 2773.5±219.86 | 2.88 |
| Fluopyram | raw cherries | 1066.36±33.82 | - |
|  | washing (1 min) | 830.84±5.64 | 0.78 |
|  | washing (2 min) | 823.44±3.45 | 0.77 |
|  | washing (5 min) | 759.74±2.72 | 0.71 |
|  | washing (10 min) | 775.6±2.28 | 0.73 |
|  | oven drying | 3507.95±99.44 | 3.29 |
|  | microwave drying | 2366.6±215.87 | 2.22 |
| Imidacloprid | raw cherries | 199.83±5.25 | - |
|  | washing (1 min) | 194.54±4.62 | 0.97 |
|  | washing (2 min) | 189.97±3.56 | 0.95 |
|  | washing (5 min) | 183.17±0.51 | 0.92 |
|  | washing (10 min) | 167.54±2.71 | 0.84 |
|  | oven drying | 892.65±47.28 | 4.47 |
|  | microwave drying | 330.43±19.7 | 1.65 |
| Kresoxim-methyl | raw cherries | 510.73±14.32 | - |
|  | washing (1 min) | 215.93±3.21 | 0.42 |
|  | washing (2 min) | 197.61±4.8 | 0.39 |
|  | washing (5 min) | 201.72±0.81 | 0.39 |
|  | washing (10 min) | 198.56±2.52 | 0.39 |
|  | oven drying | 1719.71±8.3 | 3.37 |
|  | microwave drying | 922.19±111.97 | 1.81 |
| Mefentrifluconazole | raw cherries | 626.4±15.31 | - |
|  | washing (1 min) | 562.26±3.06 | 0.90 |
|  | washing (2 min) | 527.54±2.4 | 0.84 |
|  | washing (5 min) | 492.51±2.11 | 0.79 |
|  | washing (10 min) | 465.02±5.84 | 0.74 |
|  | oven drying | 2527.38±61.9 | 4.03 |
|  | microwave drying | 2032.99±215.37 | 3.25 |
| Prochloraz | raw cherries | 767.01±17.69 | - |
|  | washing (1 min) | 702.02±10.28 | 0.92 |
|  | washing (2 min) | 667.29±2.49 | 0.87 |
|  | washing (5 min) | 602.27±9.86 | 0.79 |
|  | washing (10 min) | 554.29±6.94 | 0.72 |
|  | oven drying | 2297.81±37.17 | 3.00 |
|  | microwave drying | 2055.43±234.64 | 2.68 |
| Pyraclostrobin | raw cherries | 475.26±26.01 | - |
|  | washing (1 min) | 346.68±3.65 | 0.73 |
|  | washing (2 min) | 350.04±7.34 | 0.74 |
|  | washing (5 min) | 320.42±2.89 | 0.67 |
|  | washing (10 min) | 311.55±6 | 0.66 |
|  | oven drying | 1421.13±56.66 | 2.99 |
|  | microwave drying | 864.42±77.99 | 1.82 |
| Pyridaben | raw cherries | 1007.88±35.04 | - |
|  | washing (1 min) | 965.66±57.46 | 0.96 |
|  | washing (2 min) | 934.77±19.95 | 0.93 |
|  | washing (5 min) | 811.03±27.46 | 0.80 |
|  | washing (10 min) | 771.29±4.36 | 0.77 |
|  | oven drying | 4078.38±23.81 | 4.05 |
|  | microwave drying | 3961.65±526.73 | 3.93 |
| Spirodiclofen | raw cherries | 316.78±56.38 | - |
|  | washing (1 min) | 179.33±5.06 | 0.57 |
|  | washing (2 min) | 162.15±5.5 | 0.51 |
|  | washing (5 min) | 136.59±8.4 | 0.43 |
|  | washing (10 min) | 131.03±1.02 | 0.41 |
|  | oven drying | 981.13±3.42 | 3.10 |
|  | microwave drying | 941.85±142.96 | 2.97 |
| Tebuconazole | raw cherries | 407.73±4.76 | - |
|  | washing (1 min) | 399.06±2.07 | 0.98 |
|  | washing (2 min) | 378.19±1.59 | 0.93 |
|  | washing (5 min) | 371.51±0.68 | 0.91 |
|  | washing (10 min) | 365.38±3.32 | 0.90 |
|  | oven drying | 1365.68±35.92 | 3.35 |
|  | microwave drying | 1191.49±70.2 | 2.92 |
| Thiamethoxam | raw cherries | 754.78±25.72 | - |
|  | washing (1 min) | 709.1±12.99 | 0.94 |
|  | washing (2 min) | 683.05±13.35 | 0.90 |
|  | washing (5 min) | 689.14±13.75 | 0.91 |
|  | washing (10 min) | 610.67±8.83 | 0.81 |
|  | oven drying | 2716.82±115.52 | 3.60 |
|  | microwave drying | 678.78±31.18 | 0.90 |
| Thiophanate-methyl | raw cherries | 1292.44±49.3 | - |
|  | washing (1 min) | 206.09±5.74 | 0.16 |
|  | washing (2 min) | 175.63±4.29 | 0.14 |
|  | washing (5 min) | 143.94±3.24 | 0.11 |
|  | washing (10 min) | 135.56±3.24 | 0.10 |
|  | oven drying | 3878.74±22.24 | 3.00 |
|  | microwave drying | 1637.43±24.28 | 1.27 |
| Trifloxystrobin | raw cherries | 818.1±15.79 | - |
|  | washing (1 min) | 674.31±10.44 | 0.82 |
|  | washing (2 min) | 665.06±26.68 | 0.81 |
|  | washing (5 min) | 585.84±12.72 | 0.72 |
|  | washing (10 min) | 552.41±20.21 | 0.68 |
|  | oven drying | 2901.28±34.52 | 3.55 |
|  | microwave drying | 2369.04±188.02 | 2.90 |
| Carbendazim | raw cherries | 230.16±6.04 | - |
|  | washing (1 min) | 136.85±4.58 | 0.59 |
|  | washing (2 min) | 119.03±5.25 | 0.52 |
|  | washing (5 min) | 122.41±2.45 | 0.53 |
|  | washing (10 min) | 130.95±3.14 | 0.57 |
|  | oven drying | 1542.3±25.2 | 6.70 |
|  | microwave drying | 467.95±31.64 | 2.03 |
| Clothianidin | raw cherries | 8.88±0.11 | - |
|  | washing (1 min) | 10.93±0.22 | 1.23 |
|  | washing (2 min) | 10.01±0.46 | 1.13 |
|  | washing (5 min) | 9.39±0.12 | 1.06 |
|  | washing (10 min) | 8.35±0.22 | 0.94 |
|  | oven drying | 37.38±0.58 | 4.21 |
|  | microwave drying | 13.88±1.08 | 1.56 |

**Table S7.** The estimated daily intake (EDI), estimation of short term intake (ESTI), chronic risk quotient (RQc) and acute risk quotient (RQa) values of different pesticides for children and the adults from cherry consumption.

| Risk parameters | Pesticides | Time | | | | | | |
| --- | --- | --- | --- | --- | --- | --- | --- | --- |
|  |  | 2 h | 1 d | 3 d | 5 d | 7 d | 10 d | 14 d |
| EDI (children) | Acetamiprid | 5.8E-04 | 5.8E-04 | 5.1E-04 | 4.2E-04 | 4.0E-04 | 3.2E-04 | 1.9E-04 |
|  | Difenoconazole | 2.8E-03 | 2.4E-03 | 2.8E-03 | 2.3E-03 | 2.1E-03 | 2.0E-03 | 1.6E-03 |
|  | Fluopyram | 2.6E-03 | 2.6E-03 | 2.4E-03 | 2.1E-03 | 2.1E-03 | 2.0E-03 | 1.6E-03 |
|  | Imidacloprid | 4.8E-04 | 4.9E-04 | 4.6E-04 | 3.3E-04 | 3.2E-04 | 3.1E-04 | 1.7E-04 |
|  | Kresoxim-methyl | 1.3E-03 | 1.3E-03 | 1.1E-03 | 9.6E-04 | 8.7E-04 | 8.6E-04 | 6.6E-04 |
|  | Mefentrifluconazole | 2.0E-03 | 1.5E-03 | 1.8E-03 | 1.4E-03 | 1.2E-03 | 1.2E-03 | 1.0E-03 |
|  | Prochloraz | 1.7E-03 | 1.9E-03 | 9.9E-04 | 6.4E-04 | 2.7E-04 | 2.8E-04 | 1.4E-04 |
|  | Pyraclostrobin | 1.5E-03 | 1.2E-03 | 1.2E-03 | 1.1E-03 | 1.1E-03 | 8.8E-04 | 8.6E-04 |
|  | Pyridaben | 2.2E-03 | 2.5E-03 | 1.4E-03 | 6.6E-04 | 2.0E-04 | 1.7E-04 | 1.7E-04 |
|  | Spirodiclofen | 7.0E-04 | 7.8E-04 | 6.5E-04 | 4.4E-04 | 4.0E-04 | 3.6E-04 | 2.3E-04 |
|  | Tebuconazole | 1.1E-03 | 1.0E-03 | 1.1E-03 | 8.9E-04 | 8.0E-04 | 7.0E-04 | 4.2E-04 |
|  | Thiamethoxam | 1.9E-03 | 1.9E-03 | 1.7E-03 | 1.2E-03 | 1.3E-03 | 1.2E-03 | 6.5E-04 |
|  | Thiophanate-methyl | 2.4E-03 | 2.4E-03 | 2.4E-03 | 2.5E-03 | 2.2E-03 | 2.1E-03 | 1.8E-03 |
|  | Trifloxystrobin | 1.6E-03 | 2.0E-03 | 1.7E-03 | 1.5E-03 | 9.0E-04 | 8.9E-04 | 7.4E-04 |
|  | Clothianidin | 5.5E-06 | 2.2E-05 | 5.9E-05 | 8.8E-05 | 1.1E-04 | 1.3E-04 | 1.0E-04 |
| RQc (children) | Acetamiprid | 8.3E-06 | 8.3E-06 | 7.3E-06 | 6.1E-06 | 5.7E-06 | 4.6E-06 | 2.6E-06 |
|  | Difenoconazole | 2.8E-04 | 2.4E-04 | 2.8E-04 | 2.3E-04 | 2.1E-04 | 2.0E-04 | 1.6E-04 |
|  | Fluopyram | 2.6E-04 | 2.6E-04 | 2.4E-04 | 2.1E-04 | 2.1E-04 | 2.0E-04 | 1.6E-04 |
|  | Imidacloprid | 8.1E-06 | 8.2E-06 | 7.6E-06 | 5.5E-06 | 5.4E-06 | 5.2E-06 | 2.9E-06 |
|  | Kresoxim-methyl | 3.3E-06 | 3.2E-06 | 2.8E-06 | 2.4E-06 | 2.2E-06 | 2.1E-06 | 1.6E-06 |
|  | Mefentrifluconazole | 5.7E-05 | 4.4E-05 | 5.2E-05 | 4.0E-05 | 3.5E-05 | 3.6E-05 | 2.9E-05 |
|  | Prochloraz | 1.7E-04 | 1.9E-04 | 9.9E-05 | 6.4E-05 | 2.7E-05 | 2.8E-05 | 1.4E-05 |
|  | Pyraclostrobin | 4.9E-05 | 3.9E-05 | 4.0E-05 | 3.6E-05 | 3.6E-05 | 2.9E-05 | 2.9E-05 |
|  | Pyridaben | 2.2E-04 | 2.5E-04 | 1.4E-04 | 6.6E-05 | 2.0E-05 | 1.7E-05 | 1.7E-05 |
|  | Spirodiclofen | 7.0E-05 | 7.8E-05 | 6.5E-05 | 4.4E-05 | 4.0E-05 | 3.6E-05 | 2.3E-05 |
|  | Tebuconazole | 3.7E-05 | 3.4E-05 | 3.6E-05 | 3.0E-05 | 2.7E-05 | 2.3E-05 | 1.4E-05 |
|  | Thiamethoxam | 2.3E-05 | 2.3E-05 | 2.1E-05 | 1.5E-05 | 1.6E-05 | 1.5E-05 | 8.1E-06 |
|  | Thiophanate-methyl | 8.1E-05 | 7.8E-05 | 7.9E-05 | 8.4E-05 | 7.5E-05 | 7.0E-05 | 5.9E-05 |
|  | Trifloxystrobin | 4.0E-05 | 5.0E-05 | 4.2E-05 | 3.8E-05 | 2.3E-05 | 2.2E-05 | 1.8E-05 |
|  | Clothianidin | 5.5E-08 | 2.2E-07 | 5.9E-07 | 8.8E-07 | 1.1E-06 | 1.3E-06 | 1.0E-06 |
| EDI (adults) | Acetamiprid | 1.4E-04 | 1.4E-04 | 1.2E-04 | 1.0E-04 | 9.6E-05 | 7.7E-05 | 4.4E-05 |
|  | Difenoconazole | 6.8E-04 | 5.7E-04 | 6.7E-04 | 5.4E-04 | 4.9E-04 | 4.9E-04 | 3.8E-04 |
|  | Fluopyram | 6.2E-04 | 6.3E-04 | 5.8E-04 | 5.1E-04 | 4.9E-04 | 4.8E-04 | 4.0E-04 |
|  | Imidacloprid | 1.2E-04 | 1.2E-04 | 1.1E-04 | 8.0E-05 | 7.8E-05 | 7.5E-05 | 4.1E-05 |
|  | Kresoxim-methyl | 3.2E-04 | 3.0E-04 | 2.7E-04 | 2.3E-04 | 2.1E-04 | 2.1E-04 | 1.6E-04 |
|  | Mefentrifluconazole | 4.8E-04 | 3.7E-04 | 4.3E-04 | 3.4E-04 | 2.9E-04 | 3.0E-04 | 2.4E-04 |
|  | Prochloraz | 4.1E-04 | 4.5E-04 | 2.4E-04 | 1.5E-04 | 6.5E-05 | 6.6E-05 | 3.3E-05 |
|  | Pyraclostrobin | 3.5E-04 | 2.8E-04 | 2.9E-04 | 2.6E-04 | 2.6E-04 | 2.1E-04 | 2.1E-04 |
|  | Pyridaben | 5.2E-04 | 6.0E-04 | 3.3E-04 | 1.6E-04 | 4.8E-05 | 4.2E-05 | 4.0E-05 |
|  | Spirodiclofen | 1.7E-04 | 1.9E-04 | 1.6E-04 | 1.1E-04 | 9.7E-05 | 8.6E-05 | 5.6E-05 |
|  | Tebuconazole | 2.7E-04 | 2.4E-04 | 2.6E-04 | 2.1E-04 | 1.9E-04 | 1.7E-04 | 1.0E-04 |
|  | Thiamethoxam | 4.5E-04 | 4.5E-04 | 4.0E-04 | 2.9E-04 | 3.0E-04 | 2.8E-04 | 1.6E-04 |
|  | Thiophanate-methyl | 5.8E-04 | 5.6E-04 | 5.7E-04 | 6.0E-04 | 5.4E-04 | 5.0E-04 | 4.3E-04 |
|  | Trifloxystrobin | 3.9E-04 | 4.8E-04 | 4.1E-04 | 3.7E-04 | 2.2E-04 | 2.1E-04 | 1.8E-04 |
|  | Clothianidin | 1.3E-06 | 5.3E-06 | 1.4E-05 | 2.1E-05 | 2.5E-05 | 3.1E-05 | 2.4E-05 |
| RQc (adults) | Acetamiprid | 2.0E-06 | 2.0E-06 | 1.7E-06 | 1.5E-06 | 1.4E-06 | 1.1E-06 | 6.4E-07 |
|  | Difenoconazole | 6.8E-05 | 5.7E-05 | 6.7E-05 | 5.4E-05 | 4.9E-05 | 4.9E-05 | 3.8E-05 |
|  | Fluopyram | 6.2E-05 | 6.3E-05 | 5.8E-05 | 5.1E-05 | 4.9E-05 | 4.8E-05 | 4.0E-05 |
|  | Imidacloprid | 1.9E-06 | 2.0E-06 | 1.8E-06 | 1.3E-06 | 1.3E-06 | 1.3E-06 | 6.9E-07 |
|  | Kresoxim-methyl | 8.0E-07 | 7.6E-07 | 6.6E-07 | 5.7E-07 | 5.2E-07 | 5.2E-07 | 4.0E-07 |
|  | Mefentrifluconazole | 1.4E-05 | 1.1E-05 | 1.2E-05 | 9.6E-06 | 8.4E-06 | 8.6E-06 | 6.9E-06 |
|  | Prochloraz | 4.1E-05 | 4.5E-05 | 2.4E-05 | 1.5E-05 | 6.5E-06 | 6.6E-06 | 3.3E-06 |
|  | Pyraclostrobin | 1.2E-05 | 9.4E-06 | 9.7E-06 | 8.7E-06 | 8.7E-06 | 7.1E-06 | 6.9E-06 |
|  | Pyridaben | 5.2E-05 | 6.0E-05 | 3.3E-05 | 1.6E-05 | 4.8E-06 | 4.2E-06 | 4.0E-06 |
|  | Spirodiclofen | 1.7E-05 | 1.9E-05 | 1.6E-05 | 1.1E-05 | 9.7E-06 | 8.6E-06 | 5.6E-06 |
|  | Tebuconazole | 8.9E-06 | 8.1E-06 | 8.6E-06 | 7.2E-06 | 6.4E-06 | 5.6E-06 | 3.4E-06 |
|  | Thiamethoxam | 5.6E-06 | 5.6E-06 | 5.0E-06 | 3.6E-06 | 3.8E-06 | 3.5E-06 | 1.9E-06 |
|  | Thiophanate-methyl | 1.9E-05 | 1.9E-05 | 1.9E-05 | 2.0E-05 | 1.8E-05 | 1.7E-05 | 1.4E-05 |
|  | Trifloxystrobin | 9.6E-06 | 1.2E-05 | 1.0E-05 | 9.1E-06 | 5.4E-06 | 5.4E-06 | 4.4E-06 |
|  | Clothianidin | 1.3E-08 | 5.3E-08 | 1.4E-07 | 2.1E-07 | 2.5E-07 | 3.1E-07 | 2.4E-07 |
| ESTI (children) | Acetamiprid | 2.7E+00 | 2.7E+00 | 2.4E+00 | 2.0E+00 | 1.9E+00 | 1.5E+00 | 8.7E-01 |
|  | Difenoconazole | 1.3E+01 | 1.1E+01 | 1.3E+01 | 1.1E+01 | 9.6E+00 | 9.6E+00 | 7.4E+00 |
|  | Fluopyram | 1.2E+01 | 1.2E+01 | 1.1E+01 | 1.0E+01 | 9.6E+00 | 9.4E+00 | 7.7E+00 |
|  | Imidacloprid | 2.3E+00 | 2.3E+00 | 2.2E+00 | 1.6E+00 | 1.5E+00 | 1.5E+00 | 8.0E-01 |
|  | Kresoxim-methyl | 6.3E+00 | 5.9E+00 | 5.2E+00 | 4.5E+00 | 4.1E+00 | 4.0E+00 | 3.1E+00 |
|  | Mefentrifluconazole | 9.3E+00 | 7.3E+00 | 8.5E+00 | 6.6E+00 | 5.7E+00 | 5.9E+00 | 4.7E+00 |
|  | Prochloraz | 8.0E+00 | 8.9E+00 | 4.6E+00 | 3.0E+00 | 1.3E+00 | 1.3E+00 | 6.5E-01 |
|  | Pyraclostrobin | 6.9E+00 | 5.5E+00 | 5.7E+00 | 5.1E+00 | 5.1E+00 | 4.1E+00 | 4.0E+00 |
|  | Pyridaben | 1.0E+01 | 1.2E+01 | 6.4E+00 | 3.1E+00 | 9.4E-01 | 8.1E-01 | 7.8E-01 |
|  | Spirodiclofen | 3.3E+00 | 3.7E+00 | 3.0E+00 | 2.1E+00 | 1.9E+00 | 1.7E+00 | 1.1E+00 |
|  | Tebuconazole | 5.2E+00 | 4.7E+00 | 5.0E+00 | 4.2E+00 | 3.7E+00 | 3.3E+00 | 2.0E+00 |
|  | Thiamethoxam | 8.7E+00 | 8.7E+00 | 7.8E+00 | 5.7E+00 | 5.9E+00 | 5.5E+00 | 3.0E+00 |
|  | Thiophanate-methyl | 1.1E+01 | 1.1E+01 | 1.1E+01 | 1.2E+01 | 1.1E+01 | 9.8E+00 | 8.3E+00 |
|  | Trifloxystrobin | 7.5E+00 | 9.5E+00 | 8.0E+00 | 7.1E+00 | 4.2E+00 | 4.2E+00 | 3.5E+00 |
|  | Clothianidin | 2.6E-02 | 1.0E-01 | 2.8E-01 | 4.1E-01 | 5.0E-01 | 6.1E-01 | 4.8E-01 |
| RQa (children) | Acetamiprid | 2.7E-02 | 2.7E-02 | 2.4E-02 | 2.0E-02 | 1.9E-02 | 1.5E-02 | 8.7E-03 |
|  | Difenoconazole | 4.4E-02 | 3.7E-02 | 4.3E-02 | 3.5E-02 | 3.2E-02 | 3.2E-02 | 2.5E-02 |
|  | Fluopyram | 2.4E-02 | 2.5E-02 | 2.3E-02 | 2.0E-02 | 1.9E-02 | 1.9E-02 | 1.5E-02 |
|  | Imidacloprid | 5.7E-03 | 5.8E-03 | 5.4E-03 | 3.9E-03 | 3.8E-03 | 3.7E-03 | 2.0E-03 |
|  | Kresoxim-methyl | - | - | - | - | - | - | - |
|  | Mefentrifluconazole | 6.2E-02 | 4.8E-02 | 5.6E-02 | 4.4E-02 | 3.8E-02 | 3.9E-02 | 3.1E-02 |
|  | Prochloraz | 8.0E-02 | 8.9E-02 | 4.6E-02 | 3.0E-02 | 1.3E-02 | 1.3E-02 | 6.5E-03 |
|  | Pyraclostrobin | 1.4E-01 | 1.1E-01 | 1.1E-01 | 1.0E-01 | 1.0E-01 | 8.3E-02 | 8.0E-02 |
|  | Pyridaben | 2.0E-01 | 2.3E-01 | 1.3E-01 | 6.2E-02 | 1.9E-02 | 1.6E-02 | 1.6E-02 |
|  | Spirodiclofen | - | - | - | - | - | - | - |
|  | Tebuconazole | 1.7E-02 | 1.6E-02 | 1.7E-02 | 1.4E-02 | 1.2E-02 | 1.1E-02 | 6.5E-03 |
|  | Thiamethoxam | 8.7E-03 | 8.7E-03 | 7.8E-03 | 5.7E-03 | 5.9E-03 | 5.5E-03 | 3.0E-03 |
|  | Thiophanate-methyl | 1.1E-01 | 1.1E-01 | 1.1E-01 | 1.2E-01 | 1.1E-01 | 9.8E-02 | 8.3E-02 |
|  | Trifloxystrobin | - | - | - | - | - | - | - |
|  | Clothianidin | 4.3E-05 | 1.7E-04 | 4.6E-04 | 6.8E-04 | 8.3E-04 | 1.0E-03 | 7.9E-04 |
| ESTI (adults) | Acetamiprid | 2.6E+00 | 2.6E+00 | 2.3E+00 | 1.9E+00 | 1.8E+00 | 1.4E+00 | 8.2E-01 |
|  | Difenoconazole | 1.3E+01 | 1.1E+01 | 1.2E+01 | 1.0E+01 | 9.1E+00 | 9.1E+00 | 7.1E+00 |
|  | Fluopyram | 1.1E+01 | 1.2E+01 | 1.1E+01 | 9.5E+00 | 9.1E+00 | 9.0E+00 | 7.3E+00 |
|  | Imidacloprid | 2.1E+00 | 2.2E+00 | 2.0E+00 | 1.5E+00 | 1.4E+00 | 1.4E+00 | 7.6E-01 |
|  | Kresoxim-methyl | 5.9E+00 | 5.6E+00 | 4.9E+00 | 4.3E+00 | 3.8E+00 | 3.8E+00 | 2.9E+00 |
|  | Mefentrifluconazole | 8.8E+00 | 6.9E+00 | 8.0E+00 | 6.2E+00 | 5.4E+00 | 5.6E+00 | 4.5E+00 |
|  | Prochloraz | 7.5E+00 | 8.4E+00 | 4.4E+00 | 2.8E+00 | 1.2E+00 | 1.2E+00 | 6.2E-01 |
|  | Pyraclostrobin | 6.5E+00 | 5.2E+00 | 5.4E+00 | 4.8E+00 | 4.8E+00 | 3.9E+00 | 3.8E+00 |
|  | Pyridaben | 9.7E+00 | 1.1E+01 | 6.1E+00 | 2.9E+00 | 8.9E-01 | 7.7E-01 | 7.4E-01 |
|  | Spirodiclofen | 3.1E+00 | 3.5E+00 | 2.9E+00 | 2.0E+00 | 1.8E+00 | 1.6E+00 | 1.0E+00 |
|  | Tebuconazole | 4.9E+00 | 4.5E+00 | 4.8E+00 | 4.0E+00 | 3.6E+00 | 3.1E+00 | 1.9E+00 |
|  | Thiamethoxam | 8.3E+00 | 8.3E+00 | 7.4E+00 | 5.4E+00 | 5.6E+00 | 5.2E+00 | 2.9E+00 |
|  | Thiophanate-methyl | 1.1E+01 | 1.0E+01 | 1.0E+01 | 1.1E+01 | 1.0E+01 | 9.3E+00 | 7.9E+00 |
|  | Trifloxystrobin | 7.1E+00 | 9.0E+00 | 7.5E+00 | 6.8E+00 | 4.0E+00 | 4.0E+00 | 3.3E+00 |
|  | Clothianidin | 2.5E-02 | 9.7E-02 | 2.6E-01 | 3.9E-01 | 4.7E-01 | 5.8E-01 | 4.5E-01 |
| RQa (adults) | Acetamiprid | 2.6E-02 | 2.6E-02 | 2.3E-02 | 1.9E-02 | 1.8E-02 | 1.4E-02 | 8.2E-03 |
|  | Difenoconazole | 4.2E-02 | 3.5E-02 | 4.1E-02 | 3.3E-02 | 3.0E-02 | 3.0E-02 | 2.4E-02 |
|  | Fluopyram | 2.3E-02 | 2.3E-02 | 2.1E-02 | 1.9E-02 | 1.8E-02 | 1.8E-02 | 1.5E-02 |
|  | Imidacloprid | 5.4E-03 | 5.5E-03 | 5.1E-03 | 3.7E-03 | 3.6E-03 | 3.5E-03 | 1.9E-03 |
|  | Kresoxim-methyl | - | - | - | - | - | - | - |
|  | Mefentrifluconazole | 5.9E-02 | 4.6E-02 | 5.3E-02 | 4.1E-02 | 3.6E-02 | 3.7E-02 | 3.0E-02 |
|  | Prochloraz | 7.5E-02 | 8.4E-02 | 4.4E-02 | 2.8E-02 | 1.2E-02 | 1.2E-02 | 6.2E-03 |
|  | Pyraclostrobin | 1.3E-01 | 1.0E-01 | 1.1E-01 | 9.6E-02 | 9.7E-02 | 7.8E-02 | 7.6E-02 |
|  | Pyridaben | 1.9E-01 | 2.2E-01 | 1.2E-01 | 5.9E-02 | 1.8E-02 | 1.5E-02 | 1.5E-02 |
|  | Spirodiclofen | - | - | - | - | - | - | - |
|  | Tebuconazole | 1.6E-02 | 1.5E-02 | 1.6E-02 | 1.3E-02 | 1.2E-02 | 1.0E-02 | 6.2E-03 |
|  | Thiamethoxam | 8.3E-03 | 8.3E-03 | 7.4E-03 | 5.4E-03 | 5.6E-03 | 5.2E-03 | 2.9E-03 |
|  | Thiophanate-methyl | 1.1E-01 | 1.0E-01 | 1.0E-01 | 1.1E-01 | 1.0E-01 | 9.3E-02 | 7.9E-02 |
|  | Trifloxystrobin | - | - | - | - | - | - | - |
|  | Clothianidin | 4.1E-05 | 1.6E-04 | 4.4E-04 | 6.5E-04 | 7.9E-04 | 9.7E-04 | 7.5E-04 |
